# Autoimmune Cytopenias and Dysregulated Immunophenotype Act as Warning Signs of Inborn Errors of Immunity: Results From a Prospective Study

**DOI:** 10.3389/fimmu.2021.790455

**Published:** 2022-01-04

**Authors:** Ebe Schiavo, Beatrice Martini, Enrico Attardi, Filippo Consonni, Sara Ciullini Mannurita, Maria Luisa Coniglio, Marco Tellini, Elena Chiocca, Ilaria Fotzi, Laura Luti, Irene D’Alba, Marinella Veltroni, Claudio Favre, Eleonora Gambineri

**Affiliations:** ^1^ Department of Neurosciences, Psychology, Drug Research and Child Health (NEUROFARBA), University of Florence, Florence, Italy; ^2^ Division of Hematology, Careggi University Hospital, Florence, Italy; ^3^ Meyer University Children’s Hospital, University of Florence, Florence, Italy; ^4^ Centre of Excellence, Division of Pediatric Oncology/Hematology, Meyer University Children’s Hospital, Florence, Italy; ^5^ Division of Pediatric Oncology/Hematology, University Hospital of Pisa, Pisa, Italy; ^6^ Division of Pediatric Oncology/Hematology, University Hospital of Ospedali Riuniti, Ancona, Italy

**Keywords:** autoimmune cytopenia, autoimmune thrombocytopenia, autoimmune hemolytic anemia, autoimmune neutropenia, Evans syndrome, immunophenotyping, primary immune regulatory disorder (PIRD), inborn errors of immunity (IEIs)

## Abstract

Inborn errors of immunity (IEI) are genetic disorders characterized by a wide spectrum of clinical manifestations, ranging from increased susceptibility to infections to significant immune dysregulation. Among these, primary immune regulatory disorders (PIRDs) are mainly presenting with autoimmune manifestations, and autoimmune cytopenias (AICs) can be the first clinical sign. Significantly, AICs in patients with IEI often fail to respond to first-line therapy. In pediatric patients, autoimmune cytopenias can be red flags for IEI. However, for these cases precise indicators or parameters useful to suspect and screen for a hidden congenital immune defect are lacking. Therefore, we focused on chronic/refractory AIC patients to perform an extensive clinical evaluation and multiparametric flow cytometry analysis to select patients in whom PIRD was strongly suspected as candidates for genetic analysis. Key IEI-associated alterations causative of STAT3 GOF disease, IKAROS haploinsufficiency, activated PI3Kδ syndrome (APDS), Kabuki syndrome and autoimmune lymphoproliferative syndrome (ALPS) were identified. In this scenario, a dysregulated immunophenotype acted as a potential screening tool for an early IEI diagnosis, pivotal for appropriate clinical management and for the identification of new therapeutic targets.

## Introduction

Inborn errors of immunity (IEI) are an expanding universe of disorders, not only characterized by an infectious diathesis but also displaying a wide variety of other clinical features (1). Primary Immune Regulatory Disorders (PIRDs) are a relevant subgroup of IEI that is particularly characterized by autoimmune manifestations (2, 3). The number of genetic defects belonging to this category has strikingly expanded over time (4), and atypical manifestations of known PIRDs have progressively been unveiled (5).

In this dynamic setting, target organs of the autoimmune process may be diverse, but autoimmune cytopenias (AICs) undoubtedly play a leading role (6–8). Indeed, the relative risk of AIC appears to be at least 120 times higher in patients with IEI, compared to the general population, and increases up to 830 times if we consider autoimmune hemolytic anemia (AIHA) alone (6). Moreover, the combination of AIHA and immune thrombocytopenia (ITP) is often the clinical presentation of a PIRD (9–11), and potentially bears a genetic explanation in 65% of cases (12). Indeed, some specific immunological alterations, if accompanied with AIHA, ITP, autoimmune neutropenia (AIN), or their combinations (Evans syndrome, ES) could be significant red flags for an associated IEI (13). These include both humoral and cell-mediated immune defects, like reduced serum immunoglobulin levels and low T cell counts (3, 12, 14, 15), while only scant evidence regarding deeper immunological studies in AICs is available (16).

Regarding treatment, AICs in patients with IEI often fail to respond to first-line therapy, and the best management for refractory AICs still needs to be fully elucidated (17–20). Intravenous immunoglobulins (IVIG) and immunosuppressants are, in some cases, effective (17–19); interestingly, immunomodulatory drugs may significantly attenuate immunological alterations in PIRDs – as seen in ALPS (21, 22) – while rituximab can lead to a persistent hypogammaglobulinemia and potentially unmask an underlying genetic defect (23). Importantly, attaining a definitive molecular diagnosis might open new targeted therapeutic options, as seen in LRBA and CTLA-4 deficiencies as well as in other PIRDs (20, 24–26).

In this context, we sought to investigate the immunological and genetic background of pediatric patients affected by refractory mono- or multi-lineage AICs, eventually associated with additional signs of immune dysregulation. We applied extensive multiparametric flow cytometry, an already established tool in detecting and monitoring IEI (27, 28), to lymphocyte phenotyping on AIC background, in order to select patients in whom PIRD was suspected, and to direct next-generation sequencing (NGS) analysis. Immune phenotyping acted as a potential screening tool for an underlying IEI, thus permitting an early molecular diagnosis and a specific treatment.

## Methods

### Patient Selection and Data Collection

This prospective study included 30 pediatric and adolescent - young adult (AYA) patients (median age 8,5 years, range 1-24 years) referred to A. Meyer Children Hospital Oncology-Hematology Department for mono- or multilineage AIC, defined by immunological evaluation and/or differential diagnosis with other hematologic causes (i.e. bone marrow failure or malignancies). We recruited patients presenting with: chronic refractory ITP and/or AIHA (>12 months) requiring at least a second-line treatment; and/or AIN not self-resolving (>12 months).

According to the European Hematology Association (EHA) and the Intercontinental Childhood ITP Study (ICIS) working group criteria, ITP was defined by blood platelet count <100x10^9^/l on two separate measurements (29); AIHA by Hb level <11 g/dl and at least one hemolysis criteria among the following: reticulocytosis >120x10^9^/l, total bilirubin >1 mg/dl, and haptoglobin <10 mg/dl (30). AIN was defined as neutrophil counts <1,5x10^9^/l on two separate measurements, after excluding other secondary causes (31). Patients’ classification into two groups, isolated AIC (AIC-alone) and AIC with strong suspicion of IEI (AIC-sIEI), was based on clinical signs of immune dysregulation and/or immunodeficiency, defined as: hypogammaglobulinemia, auto/hyper-inflammation, organ-specific autoimmunity, splenomegaly, lymphadenopathy, lymphoid malignancies and/or recurrent or opportunistic infections. Eventual family history of immune disorders was considered as inclusion criteria.

First-line therapy for cytopenias included intravenous immunoglobulin (IVIG) and/or corticosteroids according to local practice; therefore, refractory ITP and AIHA were treated with second- and third-line therapy (mycophenolate mofetil, MMF; sirolimus; rituximab; eltrombopag). Due to the severity of their clinical condition at time of referral, a few patients directly underwent second- or third-line therapy. To assess response to conventional therapy, the following criteria were used: for ITP, a platelet count >30x10^9^/l with at least a two-fold increase of the pre-treatment count (29). For AIHA, Hb level ≥10 g/dl with an increase of at least 2 g/dl from baseline was considered. A response as defined above lasting at least 2 months would classify a patient as a responder (32).

**Figure 1:**
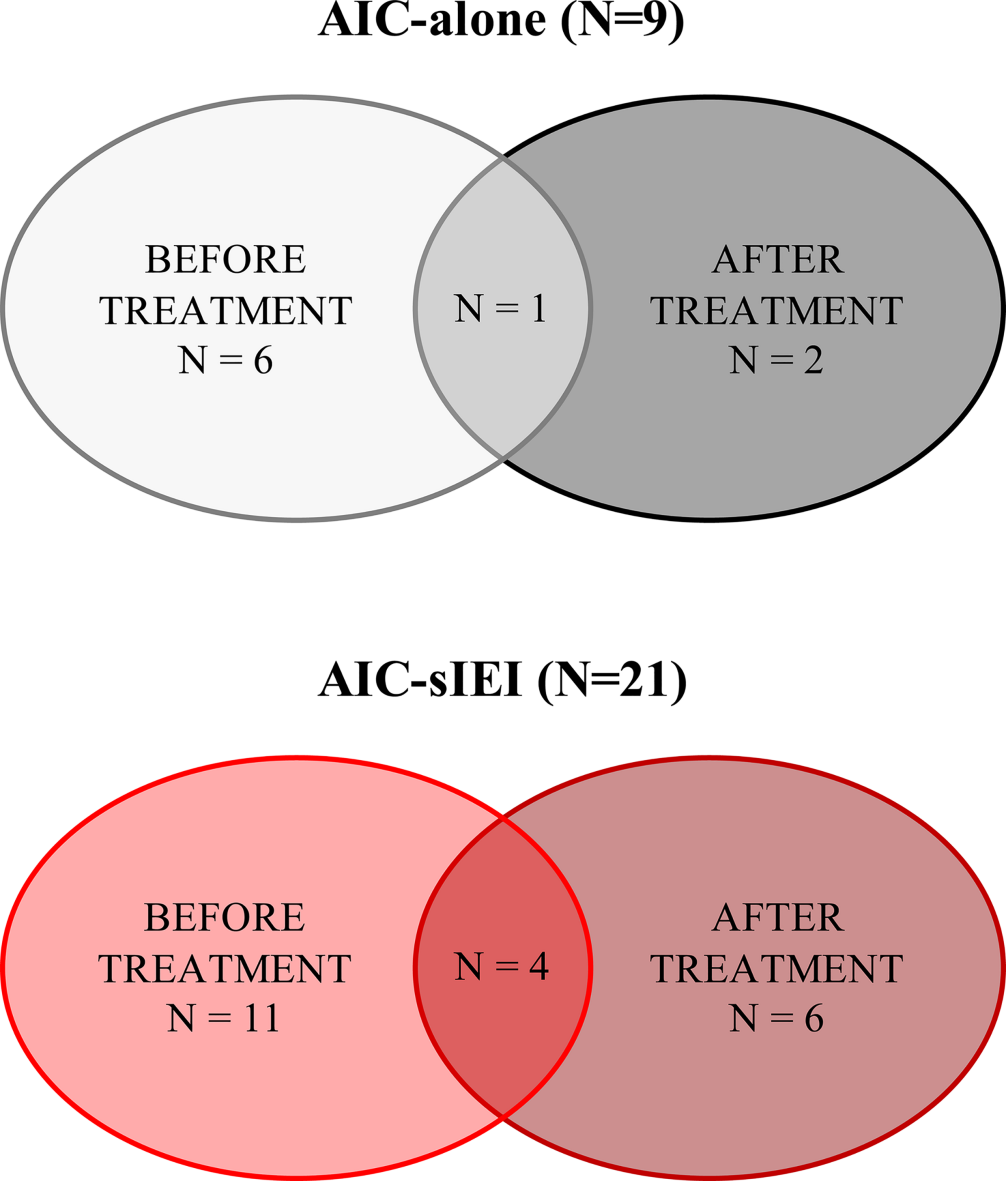
Summary of patients’ sample collection. Patients’ samples were collected before 2^nd^-line treatment (IVIG and/or corticosteroids) and/or after 2^nd^- or 3^rd^-line treatment, which included MMF, sirolimus, rituximab, and eltrombopag. N, count.

The study was reviewed and approved by Meyer Hospital Pediatric Ethics Committee, and written informed consent was obtained of all included patients or their parents, according to their age. Data related to patients’ clinical and family history was collected (**Supplementary Table 1**). Patients’ peripheral blood was analyzed at different time points to perform immunophenotyping (**Figure 1**), whereas genetic analysis was conducted on both patients and their parents in order to better interpret the genetic results.

### Immunophenotyping

Peripheral blood (PB) obtained from patients was processed within 24h after collection to perform immunophenotyping. Upon red blood cell lysis with ammonium chloride, cells were stained to identify T, B and NK cell subsets using the monoclonal antibodies listed in **Supplementary Table 2**. Flow cytometry data were collected using a MACSQuant Analyzer 10 flow cytometer (Miltenyi Biotec, Bergisch Gladbach, Germany), and analyzed with Flowlogic Software (v. 7.3, Inivai Technologies, Victoria, Australia). The expression of CD3, CD4, CD8, CD27, CD45RA, CD31 was used to identify recent thymic emigrants (RTE, CD45RA+CD31+), naïve (CD27+CD45RA+), central memory (CM, CD27+CD45RA-), effector memory (EM, CD27-CD45RA-) and terminally differentiated effector memory T cells (EMRA, CD27-CD45RA+). Treg cells were identified by CD25 and CD127 expression (CD4+CD25+CD127low), and distinguished in naïve (CD45RA+) and memory (CD45RA-) Treg. Double negative T cells (DNT) were identified by CD4 and CD8 expression within the TCRαβ T subset (TCRαβ+CD4-CD8-). The CD19+ B cell subpopulations were defined based on the differential expression of CD27 and IgD into naïve (CD27-IgD+), pre-switched memory (CD27+IgD+) and switched memory (CD27+IgD-). Plasmablasts (IgM-CD38++) and transitional B cells (CD24+CD38+) were evaluated. NK cells were defined based on CD56 expression (CD3-CD56+). A minimum of 20000 events within the lymphocyte population gate were collected, and gating strategy is shown in **Supplementary Table 3**. Absolute cell count was calculated from total lymphocyte numbers obtained by differential blood count.

### Genetic Analysis

Genetic testing was performed on the AIC-sIEI group, as patients presenting with isolated AIC (AIC-alone) did not meet the clinical and immunological criteria necessary to suspect an immunodeficit. Genomic DNA (gDNA) was extracted from peripheral blood obtained from patients and their parents using the BioRobot EZ1 Workstation (Qiagen, Milan, Italy) and quantified. Sequencing analysis was performed through target resequencing of 58 immune dysregulation-associated genes (**Supplementary Table 4**) using MiSeq Illumina platform (Illumina, San Diego, USA), or through whole-exome sequencing (WES) according to the protocols indicated. Sequence reads were aligned to the NCBI38/hg38 reference genome using a pipeline based on BWA and Picard, and variants were called using the GATK toolkit. Variants annotation (ANNOVAR tool) and prioritization was performed according to the standard guidelines of the American College of Medical Genetics and Genomics (ACMG) (33), by using a combination of prediction programs (SIFT, PolyPhen, pMUT, Mutation taster, FATHMM score, CADD score) to distinguish potentially damaging variants from those predicted to have neutral effect. Variants that were called less than 5X, off-target, synonymous, or with minor allele frequency (MAF) >1% in the Exome Aggregation Consortium (ExAC, Cambridge, MA http://exac.broadinstitute.org) were eliminated. For WES, data were filtered for a panel of >400 genes published by the International Union of Immunological Societies (IUIS) expert committee on IEI (34).

### Statistical Analysis

Analysis of lymphocyte main populations (total lymphocytes, CD3 T cells, CD4 T cells, CD8 T cells, CD19 B cells and CD56 NK cells) count (x10^9^/l) was performed using Microsoft Excel (v. 365, Microsoft Corporation, Redmond, USA), and comparisons between the two groups were made using the Student t-test (two-tailed). GraphPad Prism (v. 8.0, San Diego, USA) was used for univariate analysis of CD4, CD8, Treg and B cell subpopulation frequencies, by applying the nonparametric Mann-Whitney test (two-tailed). P values <0.05 were considered significant. Multivariate analysis on T lymphocyte subsets was performed by Principal Component Analysis (PCA), PAleontological STatistics (PAST, v. 4.03, University of Oslo). PCA is a technique for reducing the dimensionality of large datasets, minimizing information loss and increasing interpretability. The majority of the variation of flow cytometric datasets is captured by the 2 most dominant principal components (Component 1 and 2), representing a Cartesian space in which each sample (patient) is allocated. Samples are plotted to visualize similarities and differences. The overlay of the 2D (2 Dimensional) plot of the scores (patients) with the 2D plot of the loadings (combination of cell subsets) allows the identification of the variables that most contribute to the characterization of the single patient.

## Results

### Patients’ Clinical Presentation

We enrolled 30 patients, 21 males (70%) and 9 females (30%) and classified them into two groups: isolated AIC (AIC-alone) and AIC with strong suspicion of IEI (AIC-sIEI) based on the associated other clinical signs of immunodeficiency beyond AIC. Cohort clinical and laboratory features are shown in **Table 1** and **Supplementary Table 1**. The most represented cytopenia lineages are ITP and AIHA, the latter peculiar to patients with signs of immune defect. In the AIC-sIEI group, splenomegaly and hypogammaglobulinemia are the most frequent clinical signs, and almost all patients with lymphadenopathy (6/7) also presented with splenomegaly.

**Table 1:** Clinical features related to each cytopenia group.

	AIC-alone (N = 9)	AIC-sIEI (N = 21)
**General Features**		
Gender (M/F)	8/1	13/8
Median age at onset (y; range)	5; 1-15	9; 2-24
**Autoimmune Cytopenia Diagnosis (N)**		
AIHA	0	8
ITP	5	13
AIN	4	5
Trilineage cytopenia	1	4
**Immunological Features (N)**		
Family history of immune disorders	1	7
Hypogammaglobulinemia	0	8
Auto/Hyper-inflammation	0	2
Organ-specific autoimmunity	0	5
Splenomegaly	0	10
Lymphadenopathy	0	7
Malignancy	0	1
Recurrent infections	0	5
Major infections	0	1

AIHA, ITP and AIN counts include both mono- and bi-lineage cytopenia cases. N, count; y, years.

### Imbalance of Naïve and Memory T Lymphocyte Compartments in AIC Patients With Signs of Immune Dysregulation

As we aimed at defining possible congenital immune defects, causative of a wide spectrum of manifestations other than the cytopenia, we performed an extended immunophenotyping on lymphocyte subsets. Regardless of the diagnostic group, 22 patients were investigated before 2^nd^-line treatment (**Figure 1**). No significant differences concerning the absolute counts of the main immune cell populations were identified by groups comparison (**Table 2**). Absolute count of T, B and NK cells are also available for each patient (**Supplementary Table 5**).

**Table 2:** Absolute counts and frequencies of T, B and NK cell populations.

Population	AIC-alone		AIC-sIEI		*p*-value
	N	Mean (SD)	N	Mean (SD)	
Lymphocytes count (x10^9^/L)	7	2,40 (1,48)	15	2,22 (2,98)	0,86
CD3 T cells count (x10^9^/L)	7	1,66 (0,94)	15	1,02 (0,51)	0,14
CD4 Helper T cells count (x10^9^/L)	7	1,07 (0,64)	15	0,57 (0,31)	0,09
CD8 Cytotoxic T cells count (x10^9^/L)	7	0,42 (0,25)	15	0,33 (0,21)	0,42
CD19 B cells count (x10^9^/L)	7	0,31 (0,43)	13	0,95 (2,73)	0,42
CD56 NK cells count (x10^9^/L)	7	0,11 (0,09)	14	0,20 (0,18)	0,15

Mean, standard deviation (SD) and p-value of AIC-alone and AIC-sIEI group are shown. N, count.

**Figure 2:**
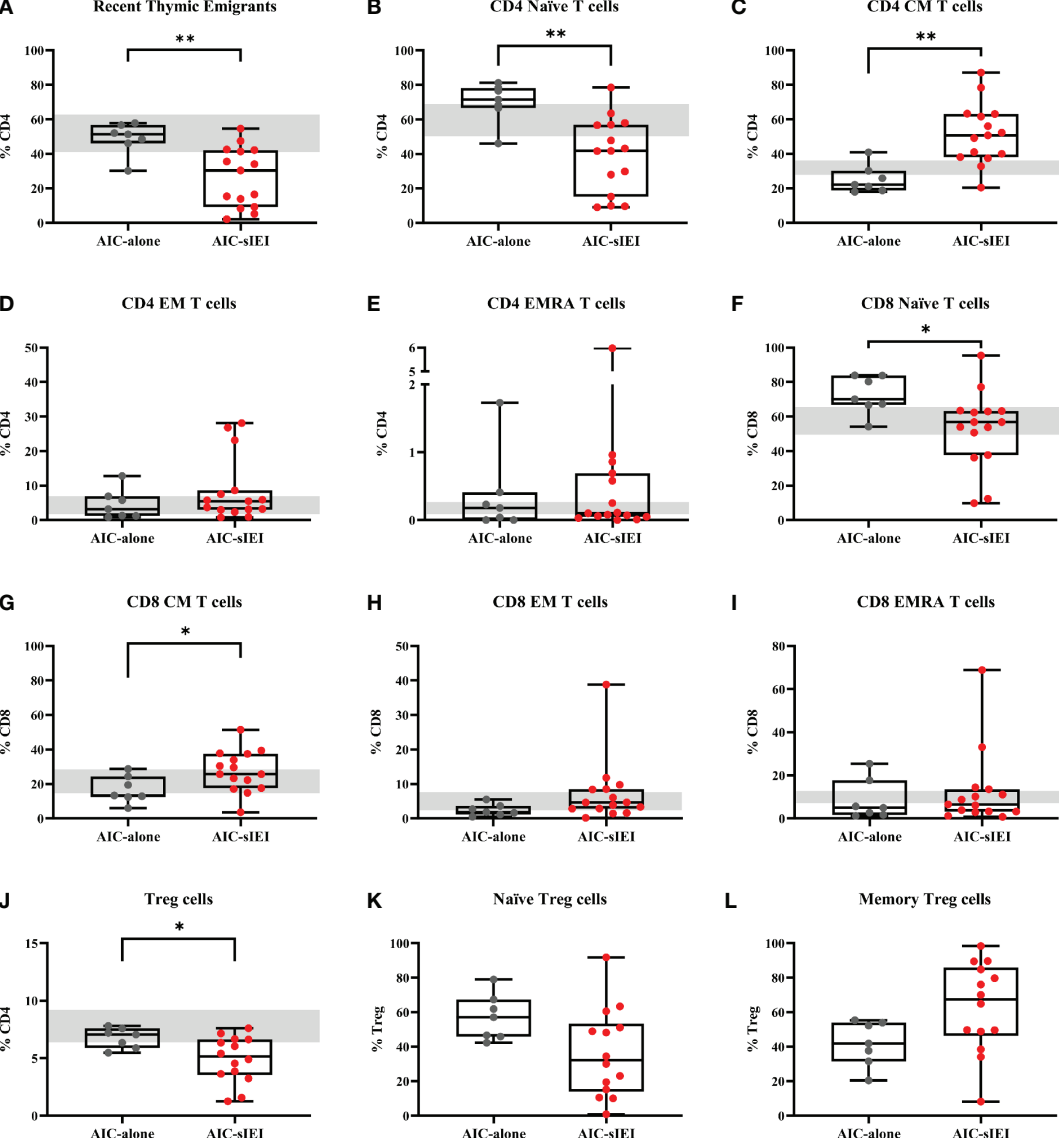
T cell subpopulations immunophenotyping analysis before 2^nd^- and 3^rd^-line treatment. Lymphocyte frequencies data (%) of AIC-alone (N=7) and AIC-sIEI (N=15) patients relative to **(A–E)** helper CD4+ T cells, **(F–I)** cytotoxic CD8+ T cells and **(J–L)** Treg subpopulations. **(L)** Treg subsets were not available for P11. Box plots show the 25^th^ percentile (bottom edge), 50^th^ percentile (median) and 75^th^ percentile (top edge); vertical lines at the top and bottom indicate minimum and maximum values. Grey bars indicate control range, based on age-matched median values (35–37). p-values <0.05 (*) or <0.01 (**) are indicated. CM, central memory T cells; EM, effector memory T cells; EMRA, terminally differentiated effector memory T cells.

Analysis performed on CD4+ T cell subsets showed significantly lower frequency of recent thymic emigrants (RTE) and naïve T cells in AIC-sIEI patients compared to AIC, with an increase of T CD4+ central memory (CM) compartment (**Figures 2A–E**). The same imbalance between naïve and memory compartments was observed for cytotoxic CD8+ T cells (**Figures 2F–I**). As Treg cells play a pivotal role in peripheral homeostasis, we also evaluated their total frequency, as well as the fraction of naïve and memory Tregs. Patients with AIC-sIEI presented a heterogeneous distribution of Treg subpopulations when compared to the AIC-only group. We also observed a reduction of total (P<0.05) and naïve Tregs, and an increase of memory Treg compartment, as detected for the other T cell lineages (**Figures 2J–L**).

**Figure 3:**
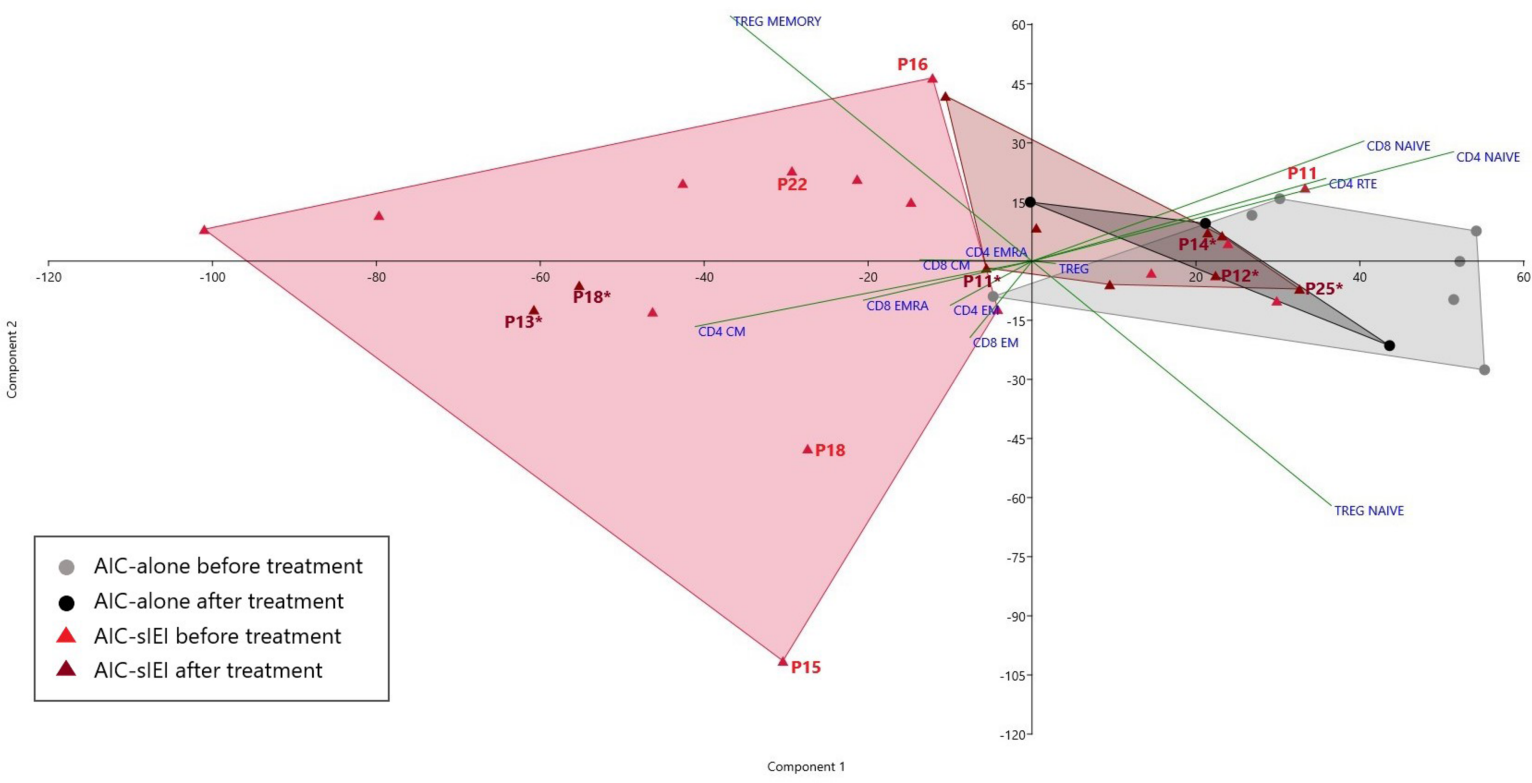
PCA of T cell subsets frequencies before and after 2^nd^- and/or 3^rd^-line treatment. Scatter plot displaying the distribution of T cell subsets frequency for AIC-alone and AIC-sIEI patients pre- and post-immunosuppressive (2^nd^- and/or 3^rd^-line) treatment (AIC-alone: N=7 before treatment, N=3 after treatment; AIC-sIEI: N=15 before treatment, N=10 after treatment). Grey and red areas indicate patients’ clusterization. AIC-sIEI patients who underwent genetic analysis are indicated for both time points (P, before treatment; P*, after treatment).

T cell subsets frequencies were then analyzed by PCA. Notably, among the AIC-sIEI group (red triangles), 11 patients out of 15 defined a specific subgroup, uniformly distributed in an area far from the AIC-only group that skewed towards CD4+ and CD8+ memory T subsets, while other 4 patients lay inside the AIC-only group area (grey dots) (**Figure 3**).

Concerning TCRαβ double negative T cell (DNT) evaluation before treatment, two patients (P14 and P18) were found to be in ALPS-range (i.e., >6% of CD3+TCRαβ+ T cells) (38), while other patients displayed borderline DNTs (**Supplementary Table 1**).

Surprisingly, we did not detect any significant difference within B cell subsets, including CD21 low B cells, by univariate (**Supplementary Figure 1**) and PCA analysis (data not shown). However, we observed very low switched memory B cell frequencies in patients with hypogammaglobulinemia, as previously described (39).

### Effects of Immunomodulatory Treatment on T Cell Subsets

Patients presenting with chronic/refractory AIC require a differential clinical management than patients with acute, transient AIC, which may need to be further adapted in presence of additional signs of immune dysregulation (13). In particular, in our cohort a higher proportion of AIC-sIEI patients underwent 2^nd^- and 3^rd^-line treatment, and for 6 patients the severity of their clinical status led to the choice of HSCT as definitive therapy. Conversely, none of the patients only presenting with AIC required HSCT, and those cases with isolated neutropenia needed no treatment (N=3) (**Table 3**).

**Table 3:** Patients’ lines of therapy.

Treatment	AIC-alone (N = 9)	AIC-sIEI (N = 21)
No treatment	3	1
1^st^-line treatment	6	15
Failed 1^st^-line treatment	4	9
2^nd^- or 3^rd^-line treatment	4	13
HSCT	0	6

AIC first-line therapy included intravenous immunoglobulin (IVIG) and/or corticosteroids; second- and third-line therapy MMF, sirolimus, rituximab, and eltrombopag. HSCT, hematopoietic stem cells transplantation; N, count.

In order to assess the treatment effect on T lymphocyte subsets, we compared immunophenotypic data obtained from AIC-alone and AIC-sIEI groups both before and after treatment with immunomodulatory agents (MMF and/or sirolimus) by PCA (**Figures 1** and **3**). Upon therapy, patients with isolated AIC did not significantly change their position in the PCA plot. On the other hand, AIC-sIEI subjects shifted towards the naïve area of the diagram, with the exception of P13 and P18 who segregated independently, suggesting a different clinical response to treatment (**Figure 3**).

### Identification of Variants in IEI-Associated Genes

Based on immunophenotyping results, we performed genetic analysis in patients with family history of immune disorders and/or signs of IEI, in order to identify the molecular bases of the observed immunological defect. Due to the advances made in sequencing technology, more than half of the patients underwent targeted NGS panel sequencing (14/21, **Supplementary Table 4**), comprising 58 genes, while in the remaining ones (7/21) WES analysis was performed (34).

**Figure 4:**
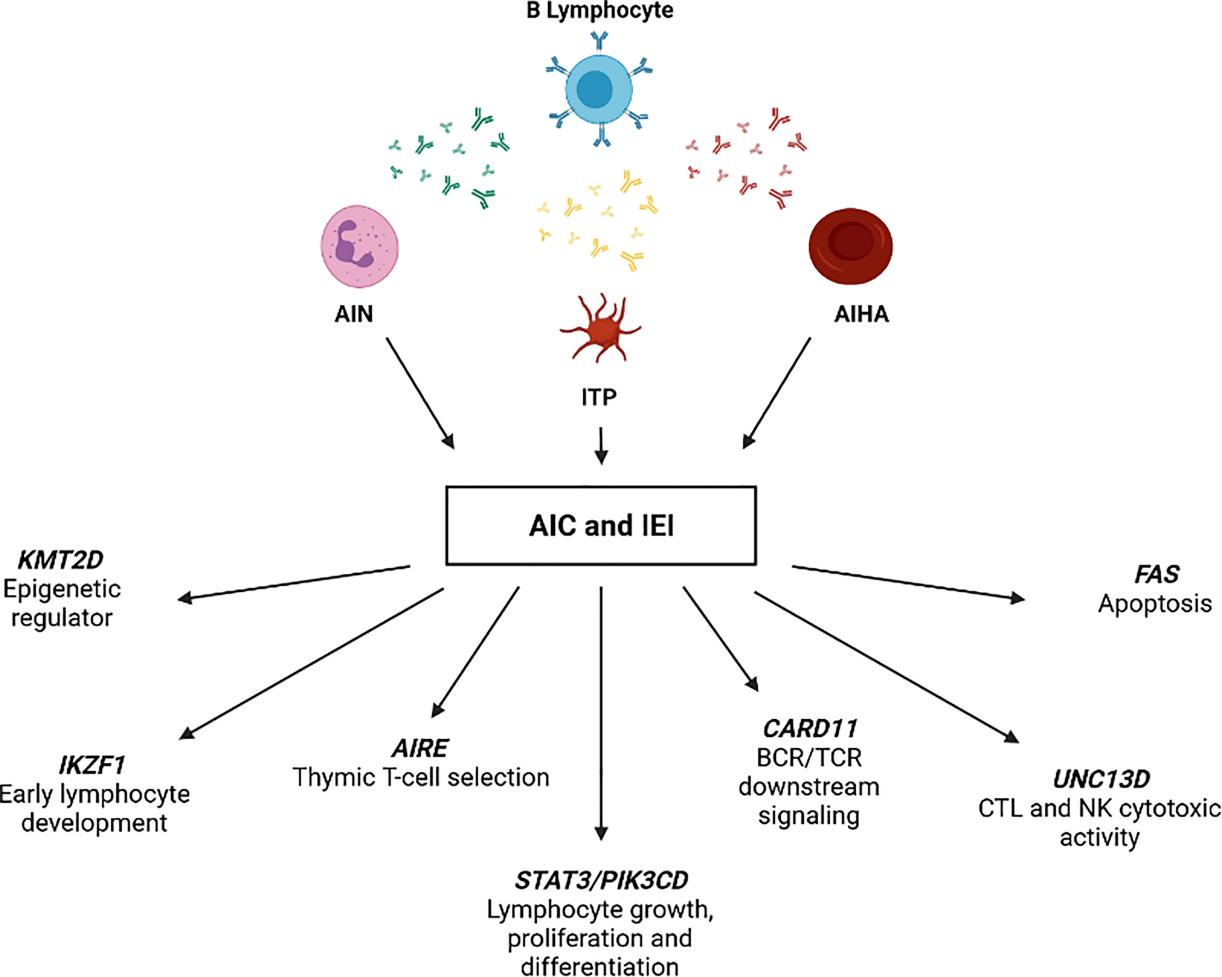
IEI-associated gene variants identified in patients with AIC and signs of immune defect (AIC-sIEI).

Strikingly, genetic analysis of 12/21 AIC-sIEI subjects was inconclusive or needed further investigation, which is currently underway. Of note, two of these (P20 and P29) had clinical and immunological features of common variable immunodeficiency (CVID). Conversely, 9/21 patients presenting with clinical features of immune dysregulation displayed disease-associated variants in the following genes (**Figure 4** and **Table 4**): *FAS, UNC13D, STAT3, CARD11, PIK3CD, KMT2D, IKZF1*, and *AIRE*.

**Table 4:** Genetic results of patients presenting with AIC associated with sings of PIRD.

Patient	Gene	OMIM and Inheritance	cDNA mutation	Zygosity	Protein mutation	HGMD Accession number	VAF	CADD	Protein function
P11	*AIRE*	240300	c.901G>A	Heterozygous	p.V301M	CM003856	<1%	25,4	LOF
	NM_000383	AD/AR							
P12	*UNC13D*	608898	c.2542A>C	Heterozygous in *cis*	p.I848L	CM137111	<1%	17,16	LOF
	NM_199242	AR	c.2983G>C		p.A995P	CM137110	<1%	14,29	
P13	*KMT2D*	147920	c.5212G>T	Heterozygous	p.E1738X	CM146820	<1%	3,5	LOF
	NM_003482	AD							
P14	*FAS*	601859	c.471_474delGACA	Heterozygous	p.T158fs	–	–	–	LOF
	NM_000043	AD							
P15	*PIK3CD*	615513	c.1574A>C	Heterozygous	p.E525A	CM1619250	<1%	26	GOF
	NM_005026	AD							
P16	*CARD11*	616452	c.1630A>C	Heterozygous	p.I544L	CM2021163	<1%	0,27	LOF
	NM_032415	AD/AR							
P18	*STAT3*	615952	c.2144C>T	Heterozygous	p.P715L	CM1713821	–	24,9	GOF
	NM_139276	AD							
P22	*UNC13D*	608898	c.3223C>T	Heterozygous	p.R1075W	VUS	<1%	9,13	LOF
	NM_199242	AR							
P25	*IKZF1*	616873	c.1505G>T	Heterozygous	p.R502L	CM212882	–	34	LOF
	NM_006060	AD							

Details on mutation, frequency (VAF, Variant Allele Frequency), CADD (Combined Annotation Dependent Depletion) score and impact on protein function are shown. CADD score integrates different genomic features such as surrounding sequence context, gene model annotations, evolutionary constraint, epigenetic measurements and functional predictions (33). VUS, Variant of Unknown Significance.

We identified a T158fs *FAS* mutation in P14, presenting with both a family history and clinical signs of autoimmune lymphoproliferative syndrome (ALPS), including high DNT frequency (21.75%) and AIHA. Coherently, he also displayed a reduced FAS expression in T cell subsets. Since other family members carried the same mutation, despite a less profound impact on protein expression, we hypothesize that P14 may also present a somatic loss of heterozygosity (sLOH) in the DNT population (40). Sanger sequencing of DNA extracted from sorted DNTs is currently ongoing.

Two loss-of-function (LOF) mutations in *UNC13D* (I848L and A995P, in *cis*) were found in P12, who clinically displayed chronic ITP and lymphoproliferation. These findings are in accordance with a previous report that considered the same *UNC13D* variants as predisposing to ALPS development (41). Interestingly, a novel variant in *UNC13D* gene (R1075W) was detected in P22, who presented a CVID-like clinical phenotype with bilineage autoimmune cytopenia (AIN+ITP) and hypogammaglobulinemia.

A *de novo* heterozygous germline *STAT3* P715L mutation previously described (42–44) was identified in P18, presenting with life-threatening AIHA and other clinical findings associated with STAT3 gain-of-function (GOF) (44, 45).

Molecular investigations performed on P16, presenting with AIHA, family history of autoimmunity, celiac disease and splenomegaly led to identification of the I544L gene variant. The variant was previously reported as benign, although autoimmune features - including cytopenias - have already been associated with hypomorphic *CARD11* mutations (46).

A known E525A *PIK3CD* mutation was detected in P15, who presented with lymphadenopathy, splenomegaly and AIHA. Based on these genetic and clinical findings, Activated PI3Kδ Syndrome (APDS) was diagnosed (47, 48).

Kabuki syndrome (KS), a rare multisystemic immune disorder, was diagnosed in P13 carrying the novel heterozygous E1738* mutation in *KMT2D* gene (49). The patient displayed typical dysmorphic features, chronic ITP and recurrent infections, which have been previously reported in other KS patients (50, 51).

A heterozygous R502L mutation in *IKZF1* gene was identified in P25, who came to our attention for Burkitt lymphoma, and subsequently developed AIN and ITP. Functional studies revealed that this genotype leads to reduced protein stability and to impaired IKAROS homo- and heterodimerization by haploinsufficiency (52).

We found the *AIRE* V301M heterozygous mutation in P11, displaying acute and persistent AIN and ITP. Homozygous *AIRE* mutations cause Autoimmune Polyendocrinopathy Candidiasis Ectodermal Dystrophy (APECED). Cytopenias have rarely been reported in APECED, even though P11 lacks other typical features and disease-specific autoantibodies (53, 54). However, heterozygous *AIRE* mutations - including V301M - may hide behind common autoimmune disorders, and lead to variable clinical manifestations among family members (55, 56).

## Discussion

This study confirms the strong relationship between AICs and IEI (13, 16), focusing on the potential role of extensive multiparametric flow cytometry and PCA as screening tools for an underlying genetic disorder. T cell phenotypes analyzed before 2^nd^- or 3^rd^-line treatment revealed an imbalanced T CD4+ and CD8+ profile in patients with AIC-sIEI. In particular, we observed a significant predominance of the mature/memory T cell compartment, counterbalanced by a reduction of T naïve and RTE subsets. Moreover, a reduced Treg frequency was detected in the AIC-sIEI group. These findings suggest the presence of an underlying immune dysregulation that skews the T cell-mediated response towards an activated status. In a clinical context, this corresponds to autoimmune features with or without lymphoproliferation, which are typically associated with PIRDs (57).

The heterogeneity of lymphocyte frequency data is in line with the high variability of IEIs that may clinically display autoimmune cytopenias (8, 13). These include CVID, which typically bears abnormal B cell subsets including a reduction in switched memory B cells (CD19+CD27+IgD-) frequency (58), especially in patients with autoimmune features (59). The scant number of CVID cases in our cohort (P20 and P29 only) may justify the lack of statistical significance in the frequencies of CD19+CD27+IgD- cells between the AIC and AIC-sIEI groups, as well as for other B cell subsets (e.g., CD21low). Interestingly, evidence suggests that the risk of autoimmunity in CVID is particularly increased in patients bearing a reduction in naïve CD4 cells, RTEs, naïve CD8 and Treg counts (60–62). These findings are surprisingly superimposable to our immunophenotyping results in the AIC-sIEI group, implying that such imbalanced T cell profile clinically correlates with autoimmunity not only in CVID but also in the entire PIRDs galaxy.

Moreover, immunophenotyping revealed elevated levels of DNT cells (38) in two patients: one affected by ALPS-FAS (P14) and the other bearing a *STAT3* GOF mutation (P18), which has recently been depicted as a possible cause of ALPS-Undetermined (ALPS-U) (63). Other patients in both groups displayed borderline DNTs, consistent with recent findings in other autoimmune contexts (64, 65). Indeed, our immunophenotypic results actually agree with an approach based on clinical and family history to select patients that should undergo molecular testing.

PCA performed on pre-treatment T cell immunophenotype showed that patients belonging to the AIC-sIEI group uniformly cluster in an area skewed towards the memory compartment (**Figure 3**), consistent with the presence of an underlying immune dysregulation. Such finding confirms the relevant role of PCA in classifying IEIs (28), and paves the way for its potential usefulness as a screening tool for patients with AICs deserving further genetic analyses. Interestingly, post-treatment PCA revealed a counter-shift of AIC-sIEI patients towards an equilibrium of naïve and memory T cell frequencies. On the other hand, treatment did not significantly impact on the position of the AIC-alone cluster in the PCA plot. This phenomenon highlights that immunomodulatory therapy (MMF and/or sirolimus) determines a partial rebalance of immune dysregulation in the AIC-sIEI subjects, consistent with a good clinical response. Therefore, these drugs might be an early treatment choice for patients with chronic/refractory AICs associated with signs of IEI, and their use should be considered according to the patient’s clinical status, as previously proposed (20). Further studies will be required to define the best therapeutic strategy for AIC patients carrying a still undiagnosed IEI.

Of note, two patients remained in the T memory area of the PCA plot after therapy: P13 and P18, respectively affected by KS and STAT3 GOF disease (49, 66). For P18, such lack of response was most likely due to the life-threatening clinical contingency that brought the patient directly to HSCT (44), without attempting targeted treatment with JAK-inhibitors and tocilizumab (26). The complex immunologic background of KS, due to an altered methylation of crucial transcription factors (51), may explain the persistence of T memory-skewed subsets in P13, which could possibly be reversed only by future applications of epigenome editing (67). Therefore, we may speculate that unbalanced immunophenotypes after immunomodulant therapy can act as a warning sign for the need, in highly selected patients, of additional treatment steps such as HSCT or - if available - targeted drugs. Further studies are needed in order to clarify this aspect.

Importantly, genetic analysis showed that IEI-causing mutations were detected in patients displaying suggestive clinical features or a positive family history (AIC-sIEI group, **Table 1**). This finding confirms previous results of a recent retrospective study (13), highlighting that associated clinical signs together with extended immunophenotyping (16) should guide physicians in the decision of performing genetic testing. Notably, 12/21 AIC-sIEI subjects had an inconclusive genetic analysis and are undergoing additional investigations, as well as P11 (*AIRE*), P16 (*CARD11*) and P22 (*UNC13D*) whose WES is currently being processed to rule out whether other mutations may cause the clinical phenotype. Given the increasing number of genes associated with IEI (1), especially within the PIRD microcosm (4), we cannot exclude that future reinterpretation of WES may unravel novel IEI-causing genotypes.

Overall, we identified several genetic causes of immune dysregulation, whose immunophenotypic behavior before and/or after immunomodulant therapy is potentially explainable. In ALPS (P14), for instance, sirolimus has already demonstrated to induce a partial normalization of biomarkers (22). On the other hand, ALPS-like disorders such as STAT3 GOF disease (P18) and APDS (P15) (68–70), as well as *CARD11* loss-of-function mutations (P16) (46), distort intracellular signaling cascades, leading to the previously described altered immunophenotype. Interestingly, hyperactivation of PI3Kδ (P15) enhances mTOR signaling, skewing the differentiation of CD8+ T cells towards short-living effector cells and impairing the development of memory T and B cells (71). Such mechanism gives a possible explanation to the peripheral position of P15 in the pre-treatment PCA plot (**Figure 3**). PI3Kδ’s pathway ultimately leads to the suppression of FOXO1, a transcription factor supporting critical genes for lymphocyte development, including IKAROS (P25, heterozygous *IKZF1* mutation) (48, 72). Nevertheless, P25’s immunophenotype after therapy shows adequate frequencies of naïve T cells - similarly to other patients with IKAROS dimerization haploinsufficiency (52). Finally, P11 (heterozygous *AIRE* mutation) displayed elevated T naïve frequencies compared to other AIC-sIEI patients, which normalized upon treatment (**Figure 3**). Interestingly, P11 presented a decrease in RTE frequencies, similar to previous reports in APECED (73, 74): such finding may potentially support the contribution of the V301M *AIRE* variant to the patient’s complex autoimmune phenotype.

Interestingly, we observed a lower frequency of AIC-alone patients in our cohort compared to recently published studies (13, 16), which could be ascribable to the different inclusion criteria and to the prospective nature of our work. Moreover, two AIC-sIEI patients presenting with multi-lineage cytopenia (P27 and P28) also displayed autoimmune hepatitis (AIH), which it is known to be associated with severe aplastic anemia (SAA). Nevertheless, an aplastic etiology was ruled out performing bone marrow aspirates and biopsies, which revealed a picture compatible with refractory cytopenia of childhood (RCC). Thus, refractory cytopenia was also recently reported as associated with autoimmune hepatitis (75). In light of their clinical behavior and immunophenotyping features, we initially interpreted these cytopenias as immune-mediated, although the clinical evolution revealed over time RCC. Therefore, we decided to include these patients in our study to raise awareness of possible overlapping hematological conditions at the time of clinical presentation.

Our real-life study has some limitations, mainly due to the restricted sample size and the scarce number of patients that underwent flow cytometry both before and after treatment. Moreover, immunophenotyping was mainly performed during acute clinical presentation, therefore we cannot exclude that these abnormalities are due to the concomitant inflammatory status, rather than the underlying immune dysregulation. However, a recent retrospective study pointed out similar immunologic alterations in patients affected by AICs with a known genetic etiology (16). Coherent findings in two differently designed studies potentially confirm that the immunologic imbalance detected in our AIC-sIEI population should not be ascribable to the concurrent inflammatory background.

**Figure 5:**
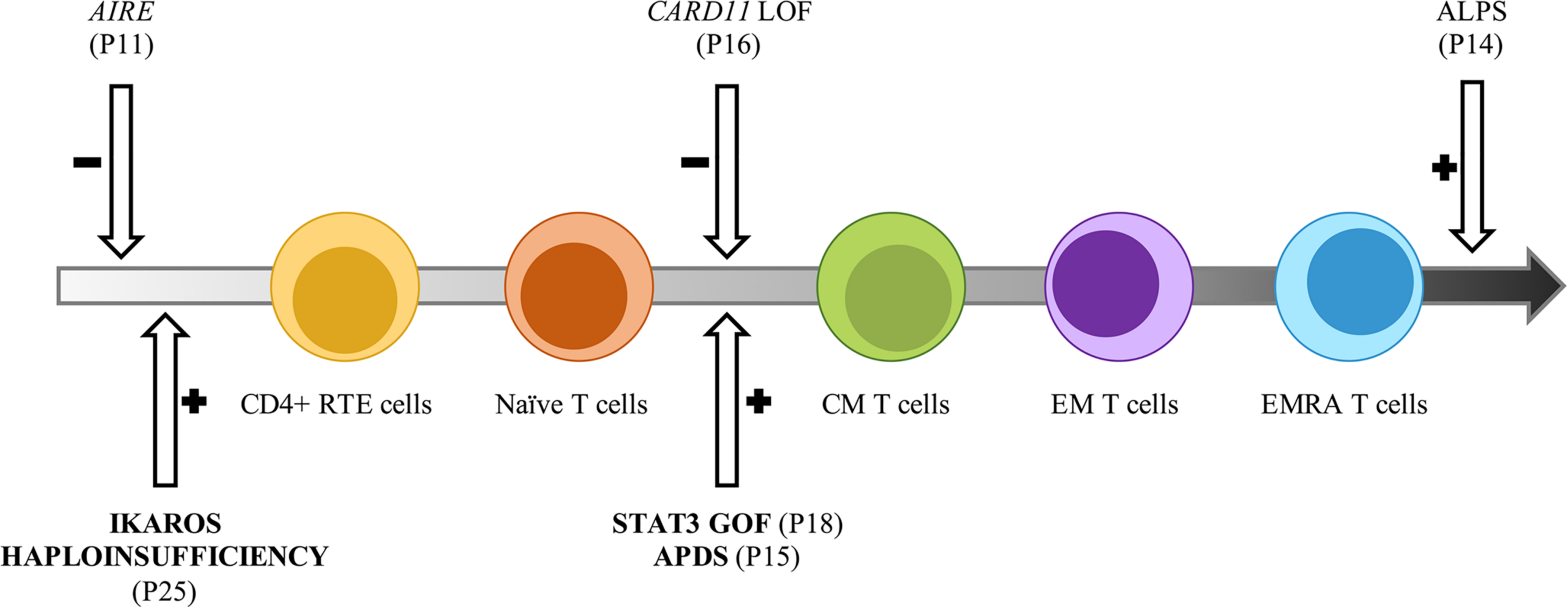
Potential impact of inborn errors of immunity on T cells development and function. Model representative of the T cell subsets alterations observed in the AIC-sIEI group. The gray arrow shows T cell populations skewed towards memory compartment and terminal effectors. Patients harboring a disease-associated (bold) or potentially relevant gene variant are indicated.

In conclusion, the tight interconnection between hematology and immunology is particularly represented by AICs, which underlie an IEI in a not negligible proportion of cases (13). This study confirms that such relationship is particularly recognizable in PIRDs and further demonstrates the kaleidoscopic presentations of IEI (3), which undoubtedly need a multidisciplinary approach. While clinical signs and family history are paramount to suspect an underlying IEI, extended immunophenotyping and PCA may potentially act as screening tools to identify patients deserving genetic analyses. In our case, patients with a strong suspicion of IEI and those who actually received a molecular diagnosis presented with T lymphocyte subsets significantly skewed towards the memory and effector compartments. Our immunophenotypic results allowed us to build a speculative model explicating how the detected genotypes may impact on specific steps of T lymphocyte’s life-cycle (**Figure 5**). Moreover, this study highlights that performing immunophenotyping before and after immunomodulatory therapy may also act as a monitor for treatment response. Larger prospective investigations are needed to improve current knowledge on clinical warning signs of IEI. Achieving a prompt diagnosis may rapidly lead to target therapies (20, 76), or definitive treatments such as HSCT or gene editing (77, 78).

## Data Availability Statement

The datasets presented in this article are not readily available because according to the protocol approved by Pediatric Ethics Committee for the current study, data sharing is limited to analysis results and not to raw datasets. Requests to access the datasets should be directed to eleonora.gambineri@unifi.it.

## Ethics Statement

The studies involving human participants were reviewed and approved by the Pediatric Ethics Committee, Meyer University Children Hospital, Florence, Italy. Written informed consent to participate in this study was provided by the participants’ legal guardian/next of kin.

## Author Contributions

ES, BM, EA, SCM, and MT performed data collection. ES, BM, and SCM performed immunophenotyping analysis. ES, BM, EA, and SCM analyzed data. MLC performed genetic analysis and analyzed data. EC, IF, LL, ID’A, MV, and EG were responsible for patient recruitment and supplied patient care. ES, BM, EA, FC, SCM, and EG wrote the original draft of the article. CF and EG supervised the work. ES and BM have contributed equally to this work and share first authorship. All authors contributed to the article and approved the submitted version.

## Funding

This work was supported by the Ministry of Health grant (Ricerca Finalizzata 2016, Ministero Della Salute RF-2016-02362384), by the Jeffrey Modell Foundation Specific Defect Research Grant (Autoimmune Cytopenias as ‘New warning sign’ of Primary Immunodeficiency Disorders) and by Ente Cassa di Risparmio di Firenze (EG).

## Conflict of Interest

The authors declare that the research was conducted in the absence of any commercial or financial relationships that could be construed as a potential conflict of interest.

## Publisher’s Note

All claims expressed in this article are solely those of the authors and do not necessarily represent those of their affiliated organizations, or those of the publisher, the editors and the reviewers. Any product that may be evaluated in this article, or claim that may be made by its manufacturer, is not guaranteed or endorsed by the publisher.
