# Supplementary material for: Autoimmune Cytopenias and Dysregulated Immunophenotype Act as Warning Signs of Inborn Errors of Immunity: Results From a Prospective Study
Source: Front Immunol. 2022 Jan 4;12:790455. doi: 10.3389/fimmu.2021.790455 (PMC8765341; doi:10.3389/fimmu.2021.790455)
Supplement: Supplementary file 1 [file Table_1.docx]

**Supplementary Table 1. Clinical features of patients enrolled in this study.** AIHA, autoimmune hemolytic anemia; AIN, autoimmune neutropenia; ITP, immune-mediated thrombocytopenia; DNT, TCR double negative T cells (CD3+TCRαβ+CD4-CD8- T cells, assessed before 2^nd^- and 3^rd^-line treatment); RA, rheumatoid arthritis; HL, Hodgkin lymphoma; BL, Burkitt lymphoma; HSCT, hematopoietic stem cells transplantation.

|  | **AIC-alone** | | | | | | | | |
| --- | --- | --- | --- | --- | --- | --- | --- | --- | --- |
| **Patient** | **P1** | **P2** | **P3** | **P4** | **P5** | **P6** | **P7** | **P8** | **P9** |
| **Gender** | M | M | M | M | M | M | F | M | M |
| **Age at onset** | 5 | 1 | 1 | 3 | 11 | 1 | 15 | 15 | 5 |
| **Cytopenia lineage** | ITP | ITP | ITP | ITP | AIN | AIN | AIN | AIN + ITP | Trilineage |
| **Family history of immune disorders** | no | no | no | Sjogren syndrome and suspect of Graves-Basedow disease (Mother); LES (maternal grandfather) | no | no | no | no | no |
| **Hypogammaglobulinemia** | no | no | no | no | no | no | no | no | no |
| **Auto/Hyper-inflammation** | no | no | no | no | no | no | no | no | no |
| **Organ-specific autoimmunity** | no | no | no | no | no | no | no | no | no |
| **Splenomegaly** | no | no | no | no | no | no | no | no | no |
| **Lymphadenopathy** | no | no | no | no | no | no | no | no | no |
| **Malignancy** | no | no | no | no | no | no | no | no | no |
| **Recurrent infections** | no | no | no | no | no | no | no | no | no |
| **Major infections** | no | no | no | no | no | no | no | no | no |
| **Other relevant features** | no | no | no | no | no | no | no | no | no |
| **DNT** | N.A. | 1,54 | N.A. | 1,25 | N.A. | 1,1 | 1,93 | 4,38 | 3,06 |
| **HSCT** | no | no | no | no | no | no | no | no | no |

**Supplementary Table 1.** (continue)

|  | **AIC-sIEI** | | | | | | | | | | |
| --- | --- | --- | --- | --- | --- | --- | --- | --- | --- | --- | --- |
| **Patient** | **P10** | **P11** | **P12** | **P13** | **P14** | **P15** | **P16** | **P17** | **P18** | **P19** | **P20** |
| **Gender** | M | M | F | M | M | F | F | M | F | M | F |
| **Age at onset** | 6 | 2 | 9 | 9 | 2 | 13 | 10 | 6 | 8 | 10 | 9 |
| **Cytopenia lineage** | ITP | ITP | ITP | ITP | AIHA | AIHA | AIHA | AIHA | AIHA + ITP | AIHA + ITP | AIHA + ITP |
| **Family history of immune disorders** | no | no | no | no | Autoimmune thyroiditis (mother); AIHA, hepato-splenomegaly,  hypergammaglobulinemia, recurrent infections (father) | no | no | no | no | Alopecia (twin brother) | Crohn syndrome and autoimmune thyroiditis (mother); Henoch-Schonlein purpura (sister) |
| **Hypogammaglobulinemia** | yes | yes | no | no | no | no | no | no | yes | yes | yes |
| **Auto/Hyper-inflammation** | no | yes | no | yes | no | no | no | no | no | no | no |
| **Organ-specific autoimmunity** | no | no | no | no | Thyroiditis | no | no | no | no | Interstitial lung disease and alopecia | no |
| **Splenomegaly** | no | no | yes | no | yes | yes | yes | no | yes | yes | no |
| **Lymphadenopathy** | no | no | yes | yes | yes | yes | no | no | yes | no | no |
| **Malignancy** | no | no | no | no | no | no | no | no | no | no | no |
| **Recurrent infections** | no | no | no | yes | no | no | no | yes | no | no | no |
| **Major infections** | no | no | no | no | HHV-7 encephalitis | no | no | no | no | no | no |
| **Other relevant features** | no | Previous AIN; rash, fever, arthralgia | no | Kabuki syndrome | Chronic skin rash | no | Atopic dermatitis | no | Growth impairment | Atopic dermatitis | CVID |
| **DNT** | 1,37 | N.A. | N.A. | N.A. | 21,75 | 2,65 | 2,54 | 1,73 | 7,71 | 1,59 | 2,41 |
| **HSCT** | no | no | no | no | no | yes | no | no | yes | no | no |

**Supplementary Table 1.** (continue)

|  | **AIC-sIEI** | | | | | | | | | |
| --- | --- | --- | --- | --- | --- | --- | --- | --- | --- | --- |
| **Patient** | **P21** | **P22** | **P23** | **P24** | **P25** | **P26** | **P27** | **P28** | **P29** | **P30** |
| **Gender** | M | M | F | M | M | M | M | F | M | F |
| **Age at onset** | 6 | 10 | 8 | 14 | 11 | 4 | 4 | 11 | 24 | 17 |
| **Cytopenia lineage** | AIHA + ITP | AIN + ITP | AIN + ITP | AIN + ITP | AIN + ITP | AIN + ITP | Trilineage | Trilineage | Trilineage | Trilineage |
| **Family history of immune disorders** | no | no | AIHA and ITP (father) | no | no | no | Psoriasis (mother, maternal grandmother, paternal grandfather) | Graves-Basedow disease (mother); RA (paternal grandfather) | HL and AIHA (father) | no |
| **Hypogammaglobulinemia** | no | yes | no | no | no | no | no | yes | yes | no |
| **Auto/Hyper-inflammation** | no | no | no | no | no | no | no | no | no | no |
| **Organ-specific autoimmunity** | no | no | no | T1 diabetes | no | no | Autoimmune hepatitis | Autoimmune hepatitis | no | no |
| **Splenomegaly** | yes | no | yes | yes | no | yes | no | no | no | no |
| **Lymphadenopathy** | yes | no | no | no | no | yes | no | no | no | no |
| **Malignancy** | no | no | no | no | BL | no | no | no | no | no |
| **Recurrent infections** | yes | no | no | no | no | yes | no | yes | no | no |
| **Major infections** | no | no | no | no | no | no | no | no | no | no |
| **Other relevant features** | Growth and language delay, facial dysmorphism, recurrent warts | no | no | Nephrotic syndrome | no | no | no | no | CVID | no |
| **DNT** | 2,16 | 4,46 | N.A. | N.A. | N.A. | 3,89 | 2,95 | 3,95 | 3,22 | 1,84 |
| **HSCT** | no | no | no | no | no | no | yes | yes | yes | yes |
