# Supplementary material for: Autoimmune Cytopenias and Dysregulated Immunophenotype Act as Warning Signs of Inborn Errors of Immunity: Results From a Prospective Study
Source: Front Immunol. 2022 Jan 4;12:790455. doi: 10.3389/fimmu.2021.790455 (PMC8765341; doi:10.3389/fimmu.2021.790455)
Supplement: Supplementary file 2 [file Table_2.docx]

**Supplementary Table 2. Monoclonal antibodies used for immunophenotyping.** Antibodies were purchased from BD Biosciences (San Jose, USA) or Miltenyi Biotec (Bergisch Gladbach, Germany).

| **Monoclonal antibody** | **Fluorochrome** | **Clone** | **Producer** |
| --- | --- | --- | --- |
| CD45 | APC-H7 | 2D1 | BD Biosciences |
| CD3 | VioGreen | REA613 | Miltenyi Biotec |
| CD4 | PerCP-Cy5.5 | SK3 | BD Biosciences |
| CD8 | VioBlue | REA734 | Miltenyi Biotec |
| CD45RA | FITC | L48 | BD Biosciences |
| CD31 | APC | AC128 | Miltenyi Biotec |
| CD27 | PE | M-T271 | Miltenyi Biotec |
| TCRαβ | APC | REA652 | Miltenyi Biotec |
| TCRγδ | PE | 11F2 | BD Biosciences |
| CD25 | PE | 2A3 | BD Biosciences |
| CD127 | AF647 | HIL-7R-M21 | BD Biosciences |
| CD45 | VioGreen | REA747 | Miltenyi Biotec |
| CD19 | PerCP-Vio 700 | REA675 | Miltenyi Biotec |
| CD27 | VioBlue | M-T271 | Miltenyi Biotec |
| CD24 | PE-Cy7 | ML5 | BD Biosciences |
| CD38 | APC-H7 | HB7 | BD Biosciences |
| CD21 | PE | HB5 | Miltenyi Biotec |
| IgM | APC | G20-127 | BD Biosciences |
| IgD | VioBright FITC | REA740 | Miltenyi Biotec |
| CD56 | VioBright 515 | REA196 | Miltenyi Biotec |
