# Supplementary material for: Autoimmune Cytopenias and Dysregulated Immunophenotype Act as Warning Signs of Inborn Errors of Immunity: Results From a Prospective Study
Source: Front Immunol. 2022 Jan 4;12:790455. doi: 10.3389/fimmu.2021.790455 (PMC8765341; doi:10.3389/fimmu.2021.790455)
Supplement: Supplementary file 3 [file Table_3.docx]

**Supplementary Table 3. Immunophenotyping gating strategy.** Lymphocyte subpopulations and gating strategy is shown. A minimum of 20000 events within the lymphocyte population gate were collected for analysis.

| **Cell population** | **Markers and gating** |
| --- | --- |
| Lymphocytes (L) | CD45+ (% WBC) |
| T cells | CD3+ (% L) |
| TCRαβ double negative T (DNT) | CD4- CD8- TCRαβ+ (% CD3+TCRαβ+) |
| CD4 helper T cells | CD3+ CD4+ (% L) |
| Recent thymic emigrants (RTE) | CD4+ CD45RA+ CD31+ (% CD4) |
| Naïve helper T cells | CD4+ CD45RA+ CD27+ (% CD4) |
| Central memory (CM) helper T cells | CD4+ CD45RA- CD27+ (% CD4) |
| Effector memory (EM) helper T cells | CD4+ CD45RA- CD27- (% CD4) |
| Terminally differentiated (EMRA) helper T cells | CD4+ CD45RA+ CD27- (% CD4) |
| CD8 cytotoxic T cells | CD3+ CD8+ (% L) |
| Naïve cytotoxic T cells | CD8+ CD45RA+ CD27+ (% CD8) |
| Central memory (CM) cytotoxic T cells | CD8+ CD45RA- CD27+ (% CD8) |
| Effector memory (EM) cytotoxic T cells | CD8+ CD45RA- CD27- (% CD8) |
| Terminally differentiated (EMRA) cytotoxic T cells | CD8+ CD45RA+ CD27- (% CD8) |
| Treg | CD4+ CD25+ CD127- (% CD4) |
| Treg naïve | CD4+ CD25+ CD127- CD45RA+ (% Treg) |
| Treg memory | CD4+ CD25+ CD127- CD45RA- (% Treg) |
| B cells | CD19+ (% L) |
| Naïve B cells | CD19+ CD27- IgD+ (% CD19) |
| Pre-switched memory B cells | CD19+ CD27+ IgD+ (% CD19) |
| Switched memory B cells | CD19+ CD27+ IgD- (% CD19) |
| Transitional B cells | CD19+ CD38+ CD24+ (% CD19) |
| CD21low | CD19+ CD38- CD21+ (% CD19) |
| Plasmablasts | CD19+ CD38++ IgM- (% CD19) |
| NK | CD56+ CD3- (% L) |
