# Supplementary material for: Autoimmune Cytopenias and Dysregulated Immunophenotype Act as Warning Signs of Inborn Errors of Immunity: Results From a Prospective Study
Source: Front Immunol. 2022 Jan 4;12:790455. doi: 10.3389/fimmu.2021.790455 (PMC8765341; doi:10.3389/fimmu.2021.790455)
Supplement: Supplementary file 5 [file Table_5.docx]

**Supplementary Table 5. Absolute counts of lymphocyte subpopulations before 2nd-line treatment.** Patients’ age and absolute numbers of white blood cells (WBC), lymphocytes, total T cells, helper T cells, cytotoxic T cells, B cells, NK cells and CD4/CD8 ratio are shown. Arrows show values above or below age-related reference values (36, 37, Mayo Clinic).

|  | **Age**  **(y)** | **WBC**  **(x10^9^/L)** | **Lympho (x10^9^/L)** | **CD3+ (x10^9^/L)** | **CD4+ (x10^9^/L)** | **CD8+ (x10^9^/L)** | **CD4/CD8**  **ratio** | **CD19+ (x10^9^/L)** | **CD56+ (x10^9^/L)** |
| --- | --- | --- | --- | --- | --- | --- | --- | --- | --- |
| **P2** | 1 | 10,46 | 4,25 | 1,76 ↓ | 0,94 ↓ | 0,70 | 1,3 | 1,28 | 0,14 |
| **P4** | 3 | 8,70 | 4,78 | 3,69 | 2,49 ↑ | 0,83 | 3,0 ↑ | 0,13 ↓ | 0,28 |
| **P5** | 12 | 3,12 ↓ | 1,56 ↓ | 1,28 | 0,81 | 0,33 ↓ | 2,5 ↑ | 0,10 ↓ | 0,11 ↓ |
| **P6** | 7 | 2,78 ↓ | 1,52 ↓ | 1,25 ↓ | 0,82 ↓ | 0,27 ↓ | 3,1 ↑ | 0,09 ↓ | 0,14 |
| **P7** | 18 | 3,12 ↓ | 1,38 | 1,17 | 0,71 | 0,37 | 1,9 ↑ | 0,09 ↓ | 0,05 ↓ |
| **P8** | 15 | 2,80 ↓ | 1,18 ↓ | 0,87 ↓ | 0,64 | 0,13 ↓ | 5,1 ↑ | 0,23 | 0,05 ↓ |
| **P9** | 5 | 2,70 ↓ | 2,11 | 1,57 | 1,05 | 0,33 ↓ | 3,2 ↑ | 0,24 | 0,02 ↓ |
| **P10** | 8 | 5,38 | 1,64 ↓ | 1,14 ↓ | 0,71 | 0,26 ↓ | 2,7 ↑ | 0,30 | 0,06 ↓ |
| **P11** | 5 | 6,32 | 1,04 ↓ | 1,01 ↓ | 0,68 | 0,31 ↓ | 2,2 ↑ | N.A. | 0,01 ↓ |
| **P15** | 13 | 8,23 | 0,89 ↓ | 0,39 ↓ | 0,14 ↓ | 0,23 ↓ | 0,6 ↓ | 0,01 ↓ | 0,32 |
| **P16** | 13 | 5,05 | 1,32 ↓ | 0,98 ↓ | 0,55 ↓ | 0,33 ↓ | 1,7 | N.A. | 0,23 |
| **P17** | 6 | 7,67 | 1,53 ↓ | 1,01 ↓ | 0,66 | 0,26 ↓ | 2,6 ↑ | 0,10 ↓ | N.A. |
| **P18** | 8 | 49,40 ↑ | 12,67 ↑ | 1,24 ↓ | 0,51 ↓ | 0,47 | 1,1 | 10,02 ↑ | 0,55 ↑ |
| **P19** | 12 | 6,41 | 2,49 | 1,65 | 1,12 | 0,44 | 2,5 ↑ | 0,47 | 0,21 |
| **P20** | 14 | 7,44 | 2,67 | 1,86 | 0,78 | 0,91 | 0,9 | 0,12 ↓ | 0,57 |
| **P21** | 10 | 7,64 | 2,63 | 1,89 | 1,10 | 0,56 | 1,9 ↑ | 0,14 ↓ | 0,36 |
| **P22** | 12 | 4,80 | 1,70 | 1,02 ↓ | 0,61 ↓ | 0,29 ↓ | 2,1 ↑ | 0,32 | 0,12 ↓ |
| **P26** | 4 | 2,90 ↓ | 1,70 ↓ | 0,83 ↓ | 0,35 ↓ | 0,26 ↓ | 1,4 | 0,57 | 0,11 ↓ |
| **P27** | 4 | 0,89 ↓ | 0,40 ↓ | 0,25 ↓ | 0,02 ↓ | 0,18 ↓ | 0,1 ↓ | 0,06 ↓ | 0,04 ↓ |
| **P28** | 11 | 2,75 ↓ | 1,49 ↓ | 1,15 | 0,65 | 0,30 ↓ | 2,2 ↑ | 0,16 | 0,14 ↓ |
| **P29** | 24 | 0,47 ↓ | 0,29 ↓ | 0,23 ↓ | 0,21 ↓ | 0,01 ↓ | 33,7 ↑ | 0,02 ↓ | 0,03 ↓ |
| **P30** | 19 | 2,72 ↓ | 0,89 ↓ | 0,70 ↓ | 0,41 ↓ | 0,16 ↓ | 2,6 ↑ | 0,04 ↓ | 0,09 ↓ |
