# Supplementary material for: Autoimmune Cytopenias and Dysregulated Immunophenotype Act as Warning Signs of Inborn Errors of Immunity: Results From a Prospective Study
Source: Front Immunol. 2022 Jan 4;12:790455. doi: 10.3389/fimmu.2021.790455 (PMC8765341; doi:10.3389/fimmu.2021.790455)

**Supplementary Figure 1. B cell subpopulations immunophenotyping analysis before 2^nd^- and 3^rd^-line treatment.** (A-D) B cell subpopulations frequency of AIC-alone (N=7) and AIC-sIEI (N=13) patients. (D) AIC-sIEI patients presenting with hypogammaglobulinemia are indicated with blue diamonds (N=8). Box plots show the 25^th^ percentile (bottom edge), 50^th^ percentile (median) and 75^th^ percentile (top edge); vertical lines at the top and bottom indicate minimum and the maximum values. Grey bars indicate control range, based on age-matched median values (37).


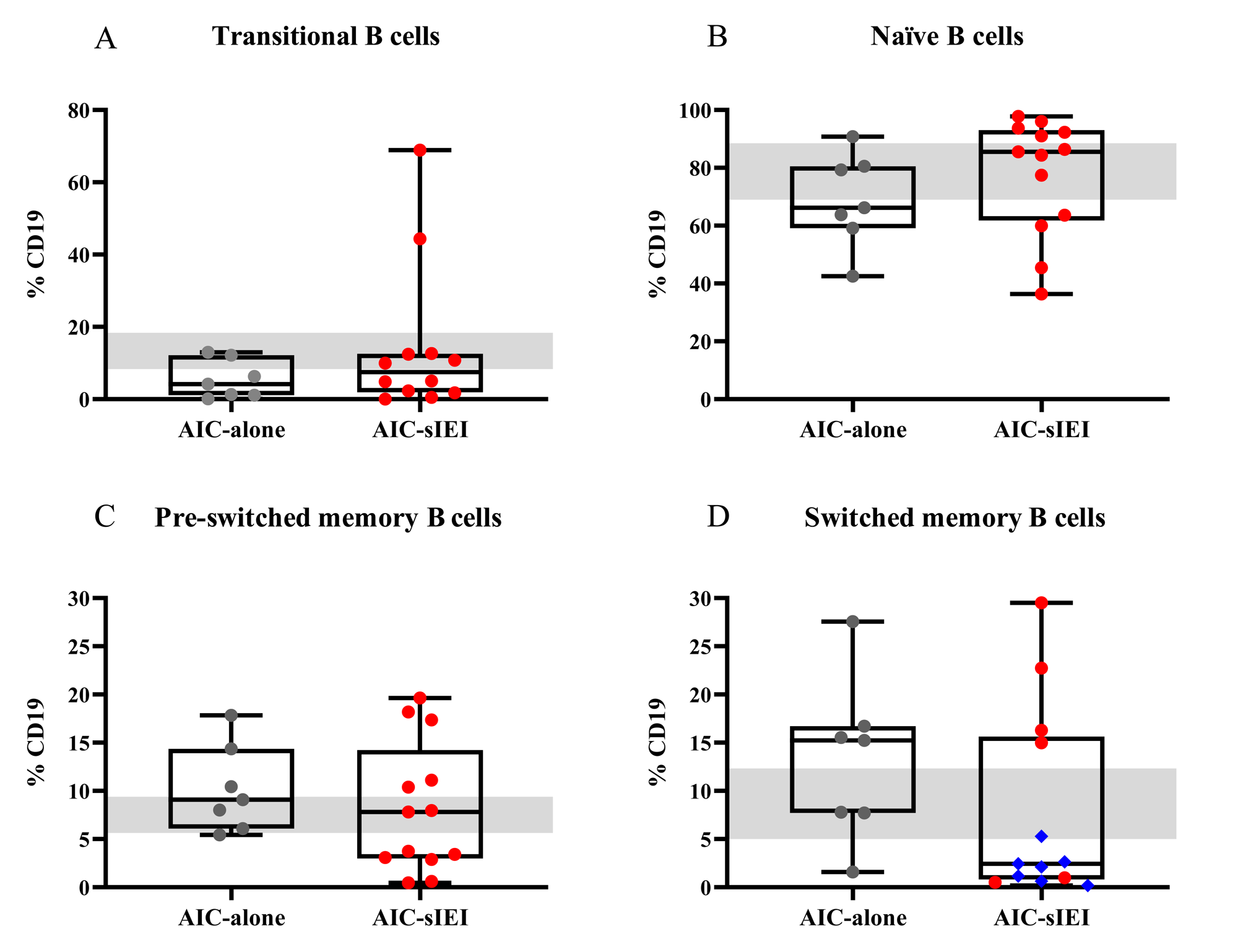

Supplement: Supplementary file 6 [file DataSheet_1.docx]
